# A Five-Year Clinical Course of Potential Phenocopy Syndrome of Behavioral Variant Frontotemporal Dementia: A Case Report and Literature Review

**DOI:** 10.7759/cureus.40118

**Published:** 2023-06-08

**Authors:** Joseph Melillo, Keyur Patel, Christian White

**Affiliations:** 1 New Jersey Institute for Successful Aging, Rowan-Virtua School of Osteopathic Medicine, Stratford, USA

**Keywords:** cognitive disorders, cognitive neuroscience, cognitive, quality of life of dementia patients, ftd, impact of dementia on older people, neuroimaging and dementia, fronto temporal dementia

## Abstract

Frontotemporal dementia is a neurocognitive disorder that affects language, behavior, or executive functioning. This disease includes a spectrum of presentations that includes multiple variants. The phenocopy syndrome of the behavioral variant of frontotemporal dementia mimics the behavioral variant of frontotemporal dementia. Patients with this condition show a decline in personality, social conduct, and cognitive ability but often display no signs of neurological imaging and exhibit slow progression. This case focuses on a now 70-year-old male who has shown signs of behavioral changes with a slowly progressive clinical course and minimal findings on positron emission tomography (PET) scan, but moderate changes seen on MRI. This report details a clinical presentation of an individual potentially with phenocopy syndrome of behavioral variant frontotemporal dementia and provides context to how symptoms can be managed to better help assist patients and their caregivers.

## Introduction

Frontotemporal dementia (FTD) is a neurodegenerative disorder that results in progressive changes in executive functioning, behavior, or language [1]. FTD encompasses three major clinical phenotypes including the behavioral variant, semantic variant of primary progressive aphasia, and nonfluent/agrammatic primary progressive aphasia [1]. Amongst these subtypes, the behavioral variant is the most common. The behavioral variant of FTD (bvFTD) is characterized by a gradual decline in personality, social conduct, and cognition. It is diagnosed by meeting three of six criteria: behavioral disinhibition, apathy, loss of empathy or sympathy, compulsive/ritualistic behaviors, hyperorality and/or dietary changes, and executive deficits with relative preservation of episodic memory and visuospatial skills [2].

In 2006, Davies et al. discovered a new subset of FTD [3]. These patients presented with similar symptoms to those of bvFTD; however, this condition was found to have slow or minimal progression in cognitive deficits [3,4]. Furthermore, this variant was characterized by unremarkable neurological imaging [3,5]. This subset of FTD was identified as the phenocopy syndrome of bvFTD (phFTD) because it appeared to “copy” the phenotype seen in bvFTD.

Executive functioning, social cognition, and episodic memory tend to be preserved in phFTD. As a result of its lack of progression and longer life expectancy, it has been commonly referred to as “benign bvFTD” [6,7]. Despite almost two decades since the identification of phFTD, the cause of this syndrome remains unclear [7].

This case focuses on a now 70-year-old male with phFTD who has been living with the diagnosis for five years, with symptoms for about 15 years. This individual showed moderate changes on MRI, but no changes on PET scan. He has shown a slow progression in symptoms since originally being diagnosed.

## Case presentation

A 66-year-old male presented to the outpatient office in 2018 with his wife reporting about 10 years of symptoms that included emotional outbursts, socially inappropriate behavior, apathy, insatiable appetite, and mild short-term memory loss. His medical history was significant for major depression, type two diabetes mellitus, hypercholesterolemia, and hypertension. He had seen a psychiatrist intermittently since 1983 for depression and was trialed on citalopram, fluoxetine, aripiprazole, clonidine, valproate, and amitriptyline. Upon reporting to the outpatient office, he was on fluoxetine HCl, bupropion HCl, and quetiapine fumarate. Family history was remarkable for both parents having unspecified dementia.

His initial physical exam was unremarkable besides the following: his effect was pleasant but defensive and disagreeing with his wife throughout which is his baseline presentation. The patient’s thought rate was slowed, his process was goal-directed and he demonstrated decreased associations. The patient’s fund of knowledge was seemingly within normal limits, but he demonstrated poor judgment and insight into the extent of his cognitive impairments.

**Figure 1:**
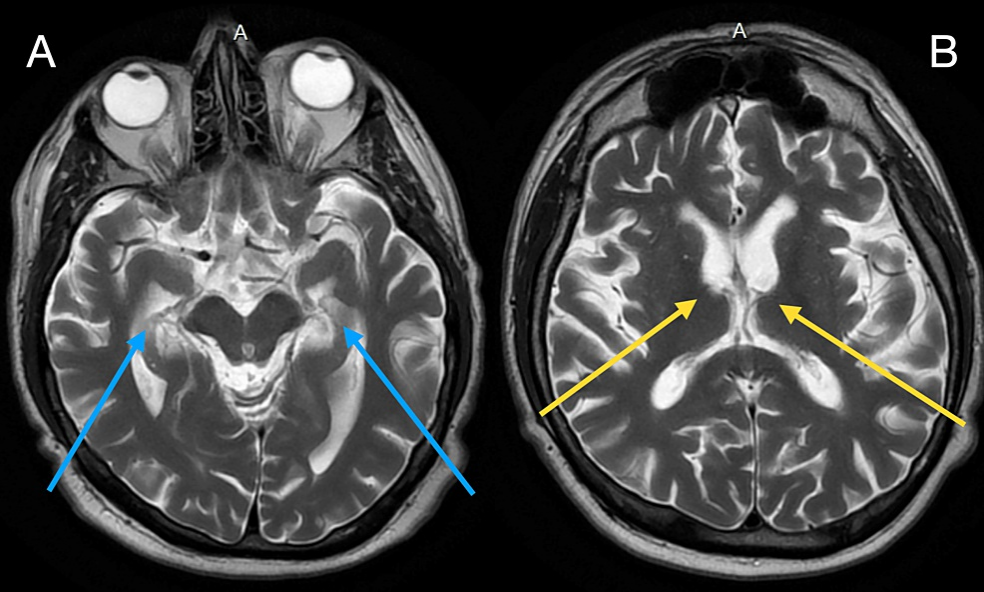
Brain MRI images of the patient MRI: Magnetic Resonance Imaging A) MRI displaying changes in hippocampal volume as depicted by the blue arrows. B) MRI showing changes in lateral ventricles volume as depicted by the yellow arrows.

An MRI was performed in late 2018 which showed a hippocampal volume of 5.56 cm^3^ belonging to the 1^st^ percentile (Figure 1A), lateral ventricles volume of 55.75 cm^3^ belonging to the 89^th^ percentile, and inferior lateral ventricles volume of 4.31 cm^3^ within the 99^th^ percentile. (Figure 1B). The impressions indicated that there was moderate generalized atrophy with some focal medial temporal volume loss with hippocampal occupancy scores being greater than two standard deviations below the mean for age-matched controls. He also displayed minimal age-related white matter change.

**Figure 2:**
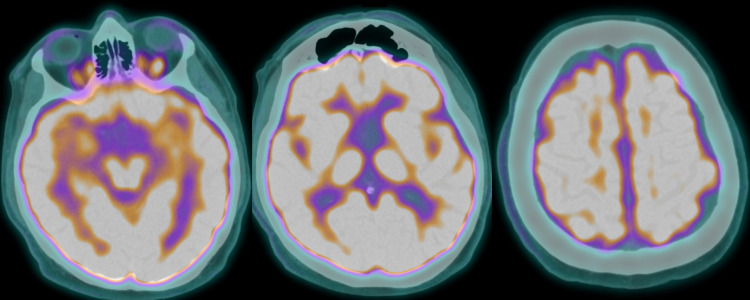
F18-FDG PET/CT scan images displaying no gross diffuse or focal metabolic abnormalities F18-FDG PET/CT: fluorine-18-fluoro-D-glucose Positron Emission Tomography/Computed Tomography

A fluorine-18-fluoro-D-glucose Positron Emission Tomography/Computed Tomography (F18-FDG PET/CT) scan was performed one month later in 2019. This imaging demonstrated increased activity within the occipital lobes, symmetric cortical activity bilaterally, preserved cerebellar activity bilaterally, and no focal abnormality in the basal ganglia or thalamus bilaterally (Figure 2). Ultimately, there were no gross diffuse or focal metabolic abnormalities.

Neuropsychiatric testing was repeated in 2019 to compare with an evaluation completed in 2018. Most domains of cognition remained steady, but he demonstrated a clinically significant decline in basic attention and encoding information. Consolidation appeared relatively intact given that he was generally able to retain what he learned. He had borderline impairments in verbal abstract reasoning and semantic fluency. Visuospatial skills were intact along with his set-shifting and cognitive inhibition. However, he made an unusually high number of errors on the inhibition task. Based on these findings, he was diagnosed with bvFTD. Despite the finding of hippocampal atrophy on MRI, which would typically indicate Alzheimer’s dementia, it was not concluded given his clinical findings, neuropsychiatric testing, and negative F18-FDG PET/CT scan. Vascular dementia was also ruled out given his lack of blood vessel abnormalities and the absence of a stepwise decline in his executive functioning.

Based on the findings of the report and the absence of emotional outbursts, quetiapine was gradually reduced and discontinued. Upon discontinuation, the patient’s wife reported increased crying spells and then a decision was made to place the patient on Nuedexta® (dextromethorphan-quinidine) 20mg-10mg every 12 hours. Nuedexta® improved the emotional outbursts, and this medication has been continued as of the present day. Escitalopram 10 mg and 20 mg were also trialed for mood lability since fluoxetine was not providing improvement. After trials of the two dosages, the treatment helped with mood, but the 20mg showed minimal to no differences from those seen on 10mg, so 10mg was maintained and continues to be taken in the present day.

Since 2019, the patient has lived away from his wife in their second home on his own and continues to do so as of the date of this report. Additionally, he still drives locally and in familiar places since he has passed multiple driving evaluations. His results on the Trail-Making Part B cognitive test have improved over the last three visits with his time on each being: 100 seconds, 90 seconds, and 55 seconds. He has been on multiple vacations both domestically and internationally since diagnosis. He was able to drive, take medications and purchase food while on an international trip in late 2022. In office visits, he has frequently been able to describe details of recent personal and current events, which his wife confirms are accurate. He has even shown the ability to learn new tasks, such as learning how to text on his cell phone.

The patient has shown some decline since his first visit in 2018. His activities of daily living are currently three out of six with deficits in bathing, continence, and toileting. He has continually shown symptoms of overconsumption of foods, making inappropriate comments about women in public, shoplifting, and bouts of anger and aggression usually towards his wife. Recently his wife reports difficulties in word finding, but minimal signs have been seen during office visits.

His last neuropsychiatric testing was completed in 2022 which demonstrated increased cognitive impairment in comparison to his prior testing done in 2019. The major neuropsychiatric symptoms found during this testing included depression, disinhibition, agitation, apathy, and irritability. His Mini Mental Status Exam (MMSE) was a 30/30, but he demonstrated minimal insight into his current cognitive impairments. When asked to copy the MMSE pentagon and produce a sentence, significant motor problems were noted. His time to complete the tasks was slower. His working memory was impaired when asked to repeat blocks of three, four, and five digits backward. The patient's memory search abilities and general intellectual functioning were also diminished, and he displayed verbal memory amnesia. Furthermore, the patient was found to have considerable dysexecutive difficulty. Ultimately the reporting suggested signs of bvFTD, however, phFTD remained a strong possibility.

Currently, the patient is still alive and is 70 years old, living in his second home independently from his wife. He continues taking escitalopram oxalate 10mg once daily, dextromethorphan-quinidine 20mg-10mg every 12 hours, and bupropion HCl 150mg once daily. Despite being unable to fulfill all his activities of daily living (ADLs) and instrumental activities of daily living (IADLs), he has shown a minimal change in baseline since the initial presentation five years ago.

## Discussion

The phenocopy syndrome of the behavioral variant of frontotemporal dementia presents similarly to the behavioral variant of frontotemporal dementia, however, the slow progression and lack of neuroimaging findings are usually key determining factors between phFTD and bvFTD. The patient presented in this report meets the criteria of phFTD regarding the slow onset of progression. This has been made evident, by his baseline remaining relatively consistent in the five years that he has been seen in the outpatient setting and has been reported to have symptoms for approximately 15 years in total. In fact, he has been able to show improvement in objective testing such as the Part B of the Trail-Making test over his last three outpatient appointments and has shown the ability to learn new tasks such as texting on a cellphone.

Typically, PET scans of FTD patients indicate hypometabolism in the frontal, anterior temporal, and anterior cingulate cortices [8]. This patient showed no signs of any metabolic changes, resembling other studies indicating a lack of neuroimaging findings in phFTD. A unique feature of this patient that is novel from the standard reporting of phFTD is the changes seen on his MRI. His MRI demonstrated atrophy of the hippocampus, which was reported to be in the first percentile for his age. There was also some medial focal temporal loss noted. The reason for the changes seen in his hippocampus remains unclear at this time. It presents neuroimaging that differs from the traditional concepts of phFTD, however, it is not the first to do so. A study conducted by Steketee et al. also found cortical brain atrophy in patients with phFTD [9]. The authors hypothesized that this finding indicates that phFTD could be a part of a spectrum of diseases within FTD [9]. The patient presented in this case provides evidence to support this thought and is a topic that requires further consideration in future research.

From the current literature, it appears that there may be an association between phFTD and other psychiatric disorders such as bipolar disorder, depression, and cluster C personality disorders [10]. Both Hornberger et al. and Bussè et al. found a correlation between depression and phFTD and suggested that it may assist in distinguishing between bvFTD and phFTD [11,12]. Our patient adds another example to the literature of an individual with suspected phFTD and a previous diagnosis of major depression. This patient had been experiencing depression since 1983. Other authors have expressed that patients with depressive symptoms often cloud providers' decision-making in diagnosing phFTD due to wanting to address this issue first as the primary diagnosis [10,12]. Future studies should consider the concurrence of other behavioral disorders such as depression as a feature in identifying phFTD.

Recent studies have investigated the associations between finances and different FTD variants and have found that patients with phFTD displayed poor financial abilities compared to healthy controls and Alzheimer’s patients with similar cognitive functioning [13]. The patient presented in this case has had trouble with spending habits but has also shown an ability to still make purchases, albeit with his limited funds. While this patient has financial limitations as compared to his previous level of functioning prior to diagnosis, he has still shown some capabilities in handling some finances as evidenced by his ability to make food purchases while on a recent vacation. Further evaluation through Financial Assessment and Capacity Test (FACT) or Financial Competence Assessment Inventory (FCAI) testing, as was done in the study conducted by Gill et al. [13], would be able to better categorize our patient’s financial capacity, however, his financial capabilities offer additional insight. His financial ability at this time also further demonstrates the slow progression that is commonly seen amongst patients with phFTD.

Limited studies in the published literature discuss the management of patients with phFTD. We are hopeful that this study detailing this patient’s use of escitalopram, dextromethorphan-quinidine, and bupropion to manage his symptoms, may help guide management for other clinicians in treating the symptoms of patients who present similarly. Based on experiences from this case, treating symptoms of phFTD appears to be the correct approach at this time. Further studies investigating different interventions for patients with phFTD would provide clarity and additional assistance in managing and treating this disorder.

In conducting a literature review, a few case reports were found on PubMed and Google Scholar detailing patients diagnosed with the phenocopy variant of frontotemporal dementia. The terms “frontotemporal dementia,” “phenocopy frontotemporal dementia,” and “behavioral variant frontotemporal dementia” were used to identify current studies. In investigating published research, limited reports detailing the progression of individuals with this condition were found. This case provides further insight into a potential clinical course seen with the phenocopy syndrome of behavioral variant frontotemporal dementia. We hope our case provides support to better understand the potential changes that can be seen in this FTD variant.

## Conclusions

This case presents a clinical summary of an individual with a possible phenocopy variant of frontotemporal dementia over the course of five years since presenting to the office. The clinical presentation depicted in this case demonstrated similar unique characteristics to others presented within the literature such as changes seen on MRI and a comorbid depression diagnosis. We hope for this case to add to the ongoing literature by providing insight into a novel presentation and management of the phenocopy variant of frontotemporal dementia.
